# Genome mining, structural elucidation and surface-active property of a new lipopeptide from *Bacillus subtilis*

**DOI:** 10.1186/s12934-025-02723-y

**Published:** 2025-05-14

**Authors:** Wan-Qi Qin, Yi-Fan Liu, Lei Zhou, Jin-Feng Liu, Dan Fei, Ke-Heng Xiang, Shi-Zhong Yang, Ji-Dong Gu, Bo-Zhong Mu

**Affiliations:** 1 State Key Laboratory of Bioreactor Engineering, School of Chemistry and Molecular Engineering, East China University of Science and Technology, Shanghai, 200237 P.R. China; 2 Shanghai Collaborative Innovation Center for Biomanufacturing Technology, Shanghai, 200237 P.R. China; 3 Daqing Huali Biotechnology Co., Ltd, Daqing, Heilongjiang 163511 P. R. China; 4 Institute of Quality Safety and Standards of Agricultural Products, Jiangxi Academy of Agricultural Sciences, Nanchang, Jiangxi 330200 P. R. China; 5 Environmental Science and Engineering Group, Guangdong Technion Israel Institute of Technology, 241 Daxue Road, Shantou, Guangdong 515063 P.R. China

**Keywords:** *Bacillus subtilis*, Genome mining, Lipopeptide, Surfactin, Surface activity

## Abstract

**Background:**

The *Bacillus* genus is well known for producing structurally diverse lipopeptides, many of which exhibit remarkable surface-active and bioactive properties, such as surfactin and daptomycin. In recent years, genome mining has emerged as an effective tool for the discovery of novel natural products by predicting biosynthetic gene clusters and linking them to secondary metabolite production. However, the full biosynthetic potential of many *Bacillus subtilis* strains remains unexplored. Therefore, this study aimed to investigate the biosynthetic potential of an oilfield-isolated *Bacillus subtilis* strain through genome mining, with the goal of identifying novel lipopeptides with enhanced surface activity.

**Results:**

In this study, we identified 14 biosynthetic gene clusters, four of which were related to lipopeptide biosynthesis. In addition, a lipopeptide was characterized as a new member of the surfactin family, namely surfactin-C18. The primary structure of surfactin-C18 was determined to be a heptapeptide ring of *N*-Glu-Leu-Leu-Val-Asp-Leu-Leu-*C* linked to the longest *β*-hydroxy fatty acid in the surfactin family, containing 18 carbon atoms. Moreover, we investigated the surface activity of surfactin-C18, measuring its critical micelle concentration and the surface tension to be 1.99 µmol/L and 28.63 mN/m, respectively. The obtained adsorption parameters of surfactin-C18 at the air/liquid interface further explained its enhanced surface activity in comparison with other surfactin homologs and commercial surfactants.

**Conclusions:**

To the best of our knowledge, this is the first report on the structural characterization and surface activity of surfactin-C18. In addition, our findings not only demonstrate the biosynthetic potential of *B. subtilis* but also highlight the power of the genome mining strategy for discovering novel lipopeptides with industrial applications.

**Supplementary Information:**

The online version contains supplementary material available at 10.1186/s12934-025-02723-y.

## Background

Lipopeptides are promising microbial-derived biosurfactants produced by various genera, including *Bacillus*, *Pseudomonas*, *Streptomyces*, *Paenibacillus*, and *Brevibacillus* [1]. Microbial lipopeptides were first discovered in the 1950s, and to date, more than 237 lipopeptides from approximately 57 families have been identified and characterized in terms of their chemical structures and properties [2]. According to their structural characteristics, microbial lipopeptides are mainly divided into three representative families: surfactin, iturin and fengycin, which typically consist of a hydrophobic fatty acid chain linked to a hydrophilic peptide chain by lactone or amide bonds [3]. This unique amphiphilic structure enables lipopeptides to modify the interfacial properties, reducing surface tension, as well as exhibiting remarkable bioactivities [4, 5]. These lipopeptides are biosynthesized by nonribosomal peptide synthetases (NRPS), which facilitate the fatty acid chain and amino acid residues incorporating into the backbone [6]. Therefore, the structural complexity and functional diversity of microbial lipopeptides highlight their potential as bioactive natural products with ever-expanding applications [7–9].

With advancements in microbial genome sequencing and bioinformatics technologies, genome mining has been considered a powerful tool to uncover biosynthetic gene clusters (BGCs) capable of encoding novel natural products [10, 11]. In particular, within the genera *Bacillus*, researchers have successfully identified new lipopeptides with previously uncharacterized structures and enhanced bioactive features by integrating genome mining with mass spectrometry detection [12]. Although hundreds of *Bacillus* genomes have been completed sequenced and uploaded into public databases, the genome mining studies remain limited in evaluating their biosynthetic potential and predicting unknown compounds [13]. Consequently, the exploration of uncharacterized BGCs and the discovery of novel bioactive natural products through genome mining in representative *Bacillus* strains remain critical research priorities.

Surfactin, primarily produced by *Bacillus* genus, has been one of the most well-known cyclic lipopeptides due to the structural diversity and excellent activities since it was first discovered in 1968 [14]. Its structure has been extensively characterized, composing of a typical heptapeptide moiety (L-Glu^1^-L-Leu^2^-D-Leu^3^-L-Val^4^-L-Asp^5^-D-Leu^6^-L-leu^7^) linked to a *β*-hydroxyl fatty acid chain of 11–17 carbon atoms to form a lactone ring structure [15]. The structural diversity of the surfactin family has been widely documented, and numerous research groups continue to isolate and characterize novel compounds, further expanding the lipopeptide family [16, 17]. In addition to its excellent surface activity under extreme environmental conditions, surfactin displays high biodegradability, low toxicity, along with reduced environmental impact [18, 19]. The outstanding physicochemical properties of surfactin also contribute to its remarkable biological activities, including antibiofilm, antimicrobial, antiviral and antitumor effects [20, 21]. These advantages have sparked increasing biotechnological and pharmaceutical interest, leading to its potential applications as biosurfactant, food additive, biopesticide and drug [22]. Therefore, it is essential to discover new surfactin variants with better performance to further enhance their applicability.

In this study, we explored the metabolic potential of a *Bacillus subtilis* strain TD7 through the genome mining approach. The presence of 14 putative biosynthetic gene clusters guided the discovery of a new lipopeptide from the surfactin family, named surfactin-C18. The compound was isolated and structurally characterized by a series of analytical techniques, including ESI-MS, LC-MS, GC-MS and NMR. Meanwhile, the surface-active property of surfactin-C18 was first preliminarily evaluated and compared with known surfactin homologs. Overall, these results laid an experimental foundation for the genome mining-based discovery of novel microbial lipopeptides, and may significantly broaden their industrial applications in enhanced oil recovery and cosmetics.

## Results and discussion

### Genomic features and annotation

We collected environmental samples from the Daqing oilfield, from which the *Bacillus subtilis* TD7 strain was isolated. To investigate the genomic and metabolic features, the complete genome of *B. subtilis* TD7 strain was sequenced, yielding a fully assembled circular chromosome (Fig. S1) with a G + C content of 43.81% (Table S1). A total of 4236 protein-coding genes (CDS) with 11 sRNAs and 30 rRNA (5–16 S-23 S rRNA) were predicted with a total length of 3,613,914 bp after genomic component analysis, which accounts for 88.77% of the full length of the genome. Comprehensive gene prediction and functional gene annotation were performed using seven commonly used databases, and a total of 4200, 3849, 3517, 3236, 2125, 4173, and 191 unique genes were matched to sequences in the NR, Swiss-Prot, Pfam, COG, GO, KEGG and CAZyme databases, respectively.

**Fig. 1:**
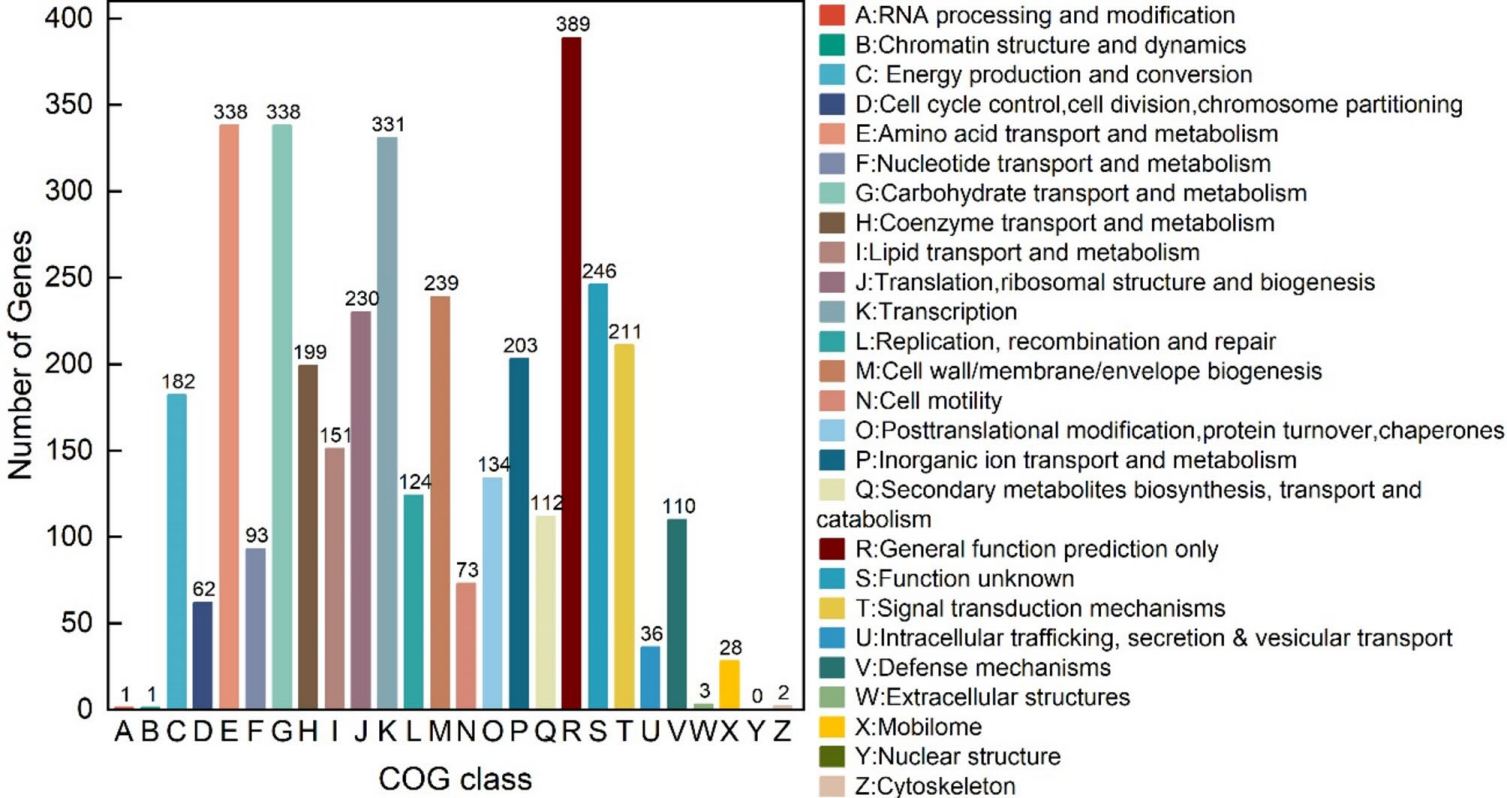
COG functional annotation classification of *B. subtilis* TD7 genome

Among the 26 functional classifications based on COG annotation analysis, the top five COG categories of *B. subtilis* TD7 were general function prediction only (R, 389 genes, 10.14%), amino acid transport and metabolism (E, 338 genes, 8.81%), carbohydrate transport and metabolism (G, 338 genes, 8.81%), transcription (K, 331 genes, 8.63%) and function unknown (S, 246 genes, 6.41%) (Fig. 1). In addition, 112 genes were successfully classified under secondary metabolites biosynthesis, transport and catabolism (S), indicating the metabolic potential of *B. subtilis* TD7. Moreover, Gene Ontology (GO) classification assigned the identified genes into 24 groups across three major functional categories: molecular function (ten groups), cellular component (five groups) and biological processes (nine groups) (Fig. S2). Among these, the genes related to biological processes were the most abundance, followed by those associated with molecular functions, while genes classified under cellular component was the least abundant. For molecular functions, the three functions with the most abundance were catalytic activity, binding and transporter activity. Among cellular components, the top classifications were membrane, membrane part and cell part. In the biological processes category, the most represented functions were metabolic process, cellular process and single-organism process. Overall, the above results indicat that many functions of *B. subtilis* TD7 are related to metabolism and membrane transport, providing robust evidence for its metabolic capabilities.

**Fig. 2:**
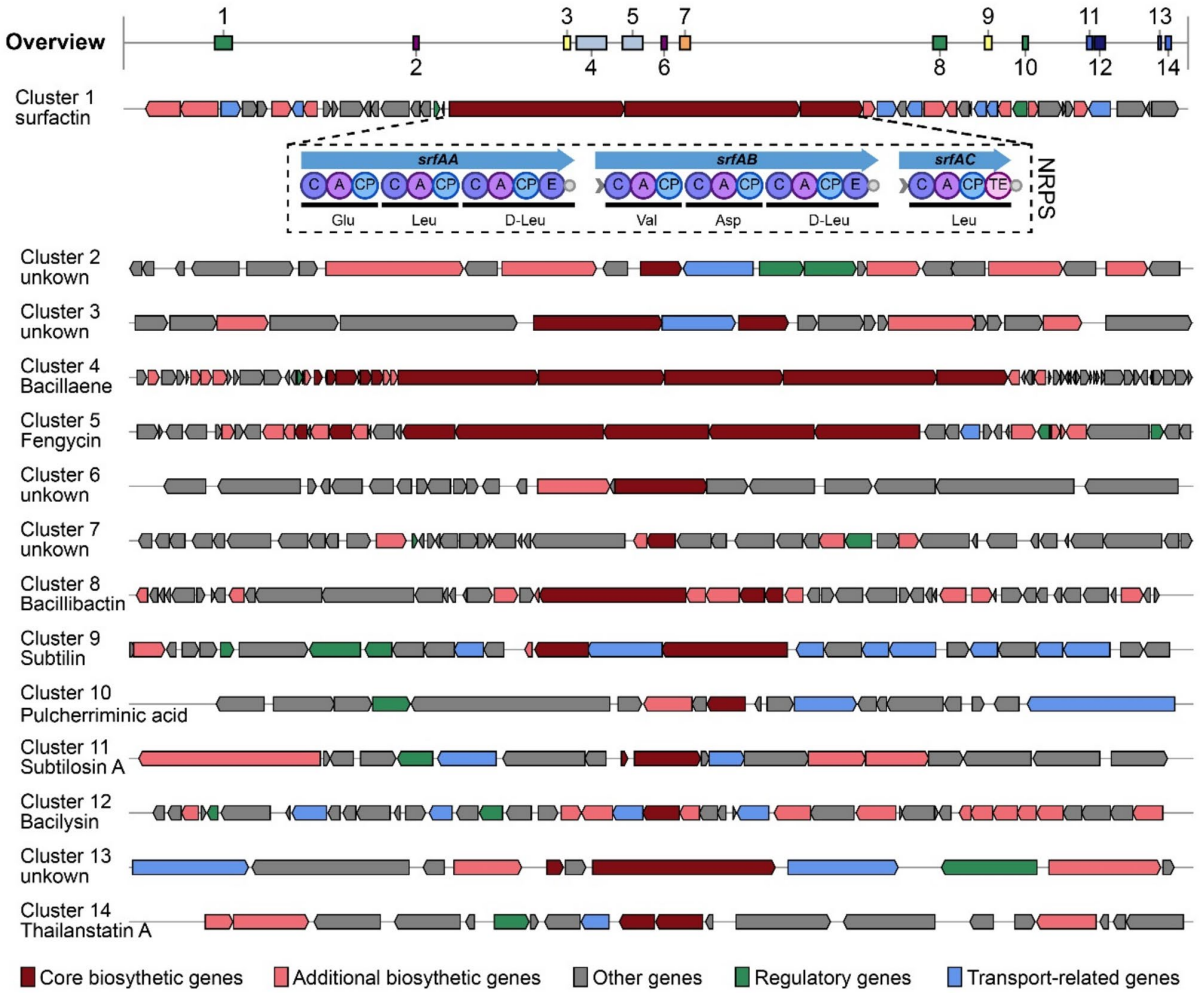
Schematic diagram of fourteen secondary biosynthesis gene clusters in *B. subtilis* TD7. The potential BGCs were predicted using antiSMASH webserver. Each number on the overview Fig. corresponds to one biosynthetic gene cluster. Genes with different functions are marked in different colour blocks, which are shown in the legend

The *Bacillus* genus is well known as a promising source of secondary metabolites, and numerous bioactive compounds, such as lipopeptides, have been extensively characterized. To better understand the metabolic characteristics of *B. subtilis* TD7, we used the antiSMASH webserver to predict biosynthetic gene clusters (BGCs) in the genome and identify their corresponding secondary metabolites. The analysis demonstrated fourteen plausible BGCs, including four nonribosomal peptide synthetases (NRPSs), one T3PKS, one lanthipeptide-class-I, one CDPS, one sactipeptide, one RiPP-like, one epipeptide, and four unclassified BGCs (Fig. 2 and Table S2). The NRPS cluster 1, which shares 82% similarity with surfactin biosynthesis in the antiSMASH database, contains all domains required for surfactin production. Thus, it is believed that the *B. subtilis* TD7 strain possesses biosynthetic potential for surfactin production.

It is commonly recognized that the rich repertoire of BGCs from the *Bacillus* genus, which encodes a diverse array of bioactive secondary metabolites, including polyketides, siderophores, terpenes, and nonribosomally lipopeptides and ribosomally synthesized peptides [23–25]. Among these, NRPS-dependent lipopeptides, such as surfactin, iturin, daptomycin, exhibit multifunctional properties as biosurfactants, antimicrobials, antibiotics and antitumor agents [26]. Given their structural diversity and functional versatility, the discovery of novel lipopeptides remains a key focus in natural product research. Therefore, using targeted genome mining tools to identify and predict BGCs responsible for the production of secondary metabolites has emerged as a powerful approach [27–29]. Here, we performed antiSMASH analysis of the *B. subtilis* TD7 genome and identified 14 putative BGCs, four of which were associated with lipopeptide biosynthesis: surfactin, fengycin, bacillaene and bacillibactin, respectively, revealing the high biosynthetic potential of the *B. subtilis* strain for producing structurally diverse lipopeptides with abundant activities.

### Isolation and purification of crude lipopeptide

Based on its unique metabolite profiles, a series of pure lipopeptide compounds have been purified from the cell-free broth via acid precipitation, ethyl acetate extraction and RP-HPLC. Several peaks were observed in the separation and preparative chromatograms of the lipopeptide extract (Fig. S3). According to our previous reports, the fractions with the elution time ranging from 15 min to 75 min corresponded to the surfactin homologs, specifically surfactin-C13 to surfactin-C17 [30]. Therefore, we speculated that the fraction at 95.6 min represented an unknown surfactin variant, which was confirmed using the ninhydrin test and TLC analysis. An analytical RP-HPLC system was subsequently employed to collect and purify this fraction for downstream analyses. The results indicated that this fraction was a pure product, designated as M1. Overall, lipopeptide M1 was collected, concentrated and subjected to a series of structural characterization techniques, with a yield of approximately 4 mg/L of culture (0.74% of total surfactin content). This yield was significantly lower than that of other surfactin homologs, as determined by LC-MS analysis as described previously (Table 1) [31]. Considering the minor differences in the molecular configuration and composition among surfactin homologs, there is no consistent retention time pattern for surfactins with the same or different *β*-hydroxy fatty acid chain lengths, especially when analyzing mixtures of homologs with varying fatty acid chain lengths. To address this challenge, Zhao et al. developed a new GC-MS method for the accurate quantification of individual surfactin homologs based on their *β*-hydroxy fatty acid lengths [32]. After hydrolysis and methyl esterification, the extracted ion chromatogram at *m/z* = 103 was used to identify *β*-hydroxy fatty acids. Furthermore, differences in molecular configurations and lengths resulted in variation in retention time, even for *β*-OH fatty acid methyl esters of the same chain lengths. Using this method, Meng et al. successfully compared the composition and relative abundance of different surfactin homologs produced by wild-type and mutan *B. subtilis* strains [33].

**Table 1:** Yield and composition of different surfactin homologs produced by *B. subtilis* TD7

Surfactin homologs	Yield (mg/L)	Proportion (%)
Surfactin-C12	23.24 ± 1.09	4.32 ± 0.29
Surfactin-C13	138.23 ± 5.39	26.02 ± 1.97
Surfactin-C14	139.47 ± 5.40	25.56 ± 0.64
Surfactin-C15	147.97 ± 6.72	27.13 ± 0.88
Surfactin-C16	64.74 ± 4.80	12.01 ± 0.55
Surfactin-C17	19.70 ± 0.61	3.66 ± 0.16
Surfactin-C18	4.00 ± 1.91	0.74 ± 0.34

### Structural characterization

**Fig. 3:**
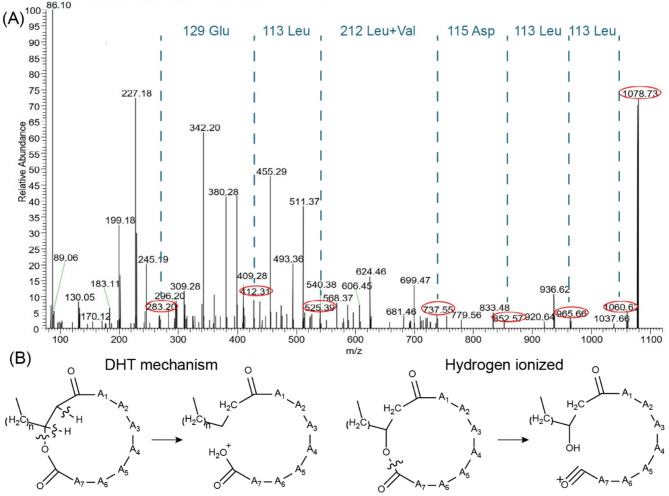
(**A**) Mass spectrogram of hydrogen ionized lipopeptide M1 and their hydrogen ionized fragments. (**B**) Cleavage of lipopeptide M1 according to the double hydrogen transfer mechanism of aliphatic esters and simple cleavage

Lipopeptide M1 was obtained as a yellowish-brown solid. The molecular mass of lipopeptide M1 was determined by high-resolution electrospray ionization mass spectrometry (ESI-MS) analysis in the positive ionization mode (Fig. 3). The most abundant fragment ion was at *m/z* 1078.73 [M + H]^+^, which was preliminarily attributed to the surfactin family of lipopeptides. According to previously reported studies, the molecular weight of surfactin-C17 (*m/*z 1063.06) was 14 Da less than that of lipopeptide M1, corresponding to an additional methylene group. These observed main peaks (*m/z* values) also corresponded to various surfactin homologs containing different types of amino acids or amino acid modifications at different locations on the peptide ring [34]. Therefore, further analysis of the chemical structure of the fatty acid chain and peptide ring is required.

The detailed information on the chemical structure of lipopeptide M1 was based on ESI-MS (Fig. 3A). A traditional method used to determine the sequence of amino acids in lipopeptide is Edman degradation, which relies on analyzing separated fragments obtained after partial hydrolysis of the cyclic lipopeptide [35]. However, Yang et al. established a TOF MS/MS method to directly determine the relationship of amino acid residues without hydrolysis [36]. According to the double hydrogen transfer (DHT) mechanism, a type of the McLafferty rearrangements of alkoxy group of aliphatic esters and amides, this process was considered a diagnostic tool in mass spectrometry to confirm the presence of carbonyl-containing functional group [37]. Thus, taking the ion peak *m/z* at 1078.73 as a precursor, the observed loss of 18 Da from 1078.73 to 1060.62 was assigned to a ring-opening reaction of an ester bond in the cyclic lipopeptide. Additionally, based on the simple cleavage of the hydrogen-ionized molecular ions, several fragment ions corresponded to the loss of amino acid residues (Fig. 3B). The fragment ions at *m/z* 1060.62, 965.57, 737.55, 525.39, 412.31 and 283.20 from lipopeptide M1 suggested the following amino acid sequence: *N*-Glu-Leu-Leu-Val-Asp-Leu-Leu-*C* (Table 2). The amino acid sequence of lipopeptide M1 was further verified after trimethylsilylation via gas chromatography-mass spectrometer (GC-MS) analysis. Compared with the standard amino acid sample, the result indicated the presence of leucine (Leu), aspartic acid (Asp), glutamic acid (Glu) and valine (Val) (Fig. S4). The peak area of each identified amino acid was normalized to determine the molar ratio of Leu: Asp: Glu: Val as 4: 1: 1: 1. The proposed amino acid sequence of the lipopeptide M1, *N*-Glu-Leu-Leu-Val-Asp-Leu-Leu-*C*, was consistent with the structure of a typical surfactin [38].

**Table 2:** Intensive ion peaks observed for surfactin and possible compositions

Fragment peaks (m/z)	Possible composition
1078.73	M + H^+^
Fragments after DHT	
1060.62	M + H^+^ - OH_2_
245.10	Leu + Leu + H^+^ + OH_2_
130.05	Leu + OH_2_
Fragments after simple cleavage	
965.66	FA residue + Glu + Leu + Leu + Val + Asp + Leu + H^+^
852.57	FA residue + Glu + Leu + Leu + Val + Asp + H^+^
737.55	FA residue + Glu + Leu + Leu + Val + H^+^
568.37	Glu + Leu + Leu + Val + Asp + H^+^
525.39	FA residue + Glu + Leu + H^+^
455.29	Glu + Leu + Leu + Val + H^+^
412.31	FA residue + Glu + H^+^
283.20	FA residue + H^+^

**Fig. 4:**
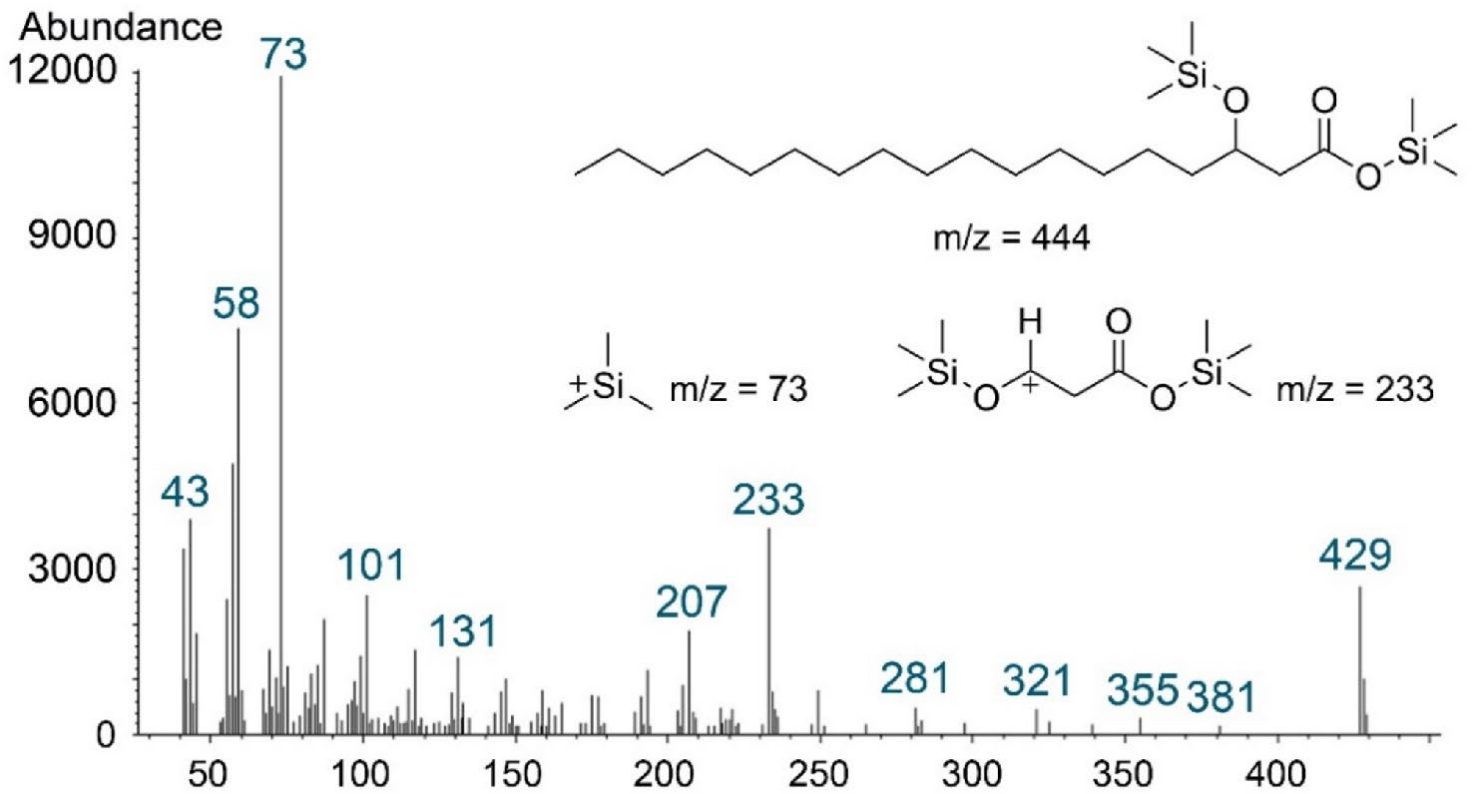
GC-MS spectrum of fatty acid residue by trimethylsilylation from lipopeptide M1

After hydrolyzed into free fatty acid and trimethylsilylation derivatization, the trimethylsilylation method was used to evaluate the hydrophobic part of lipopeptide M1 with GC-MS (Fig. 4). The BSTFA (*N*, *O*-bis(trimethylsilyl)-trifluoroacetamide) reacted with the fatty acid part of the original surfactin sample, which consisted of *β*-hydroxyl fatty acid replacing active hydrogens with -Si(CH_3_)_3_. The characteristic peak of *m/z* = 233 suggested that there was a structure of -[CHO(Si(CH_3_)_3_)CH_2_COOSi(CH_3_)_3_]^+^, replacing one hydrogen and one methyl on the -[CH(OH)CH_2_COOCH_3_]^+^ (*m/z* = 103) with - Si(CH_3_)_3_ group, which is the characteristic fragmentation ion of a *β*-hydroxy fatty acid. Since the peak of *m/z* = 429 corresponded to the molecular mass of the analyzed fatty acid part, the presence of this peak confirmed the structure of the fatty acid chain with 18 carbon atoms. ^13^C NMR spectroscopy is a direct way applied to determine the structures of fatty acid variations in lipopeptide (*iso*, *anteiso* and *normal*). In addition to NMR, the structure of the fatty acid chains in cyclic lipopeptide can be easily determined via MS spectrogram according to the ratio of the relative intensity of I_43_/I_57_ [39]. The relatively larger, smaller and middle values correspond to *iso-*, *anteiso*- and *n*-, respectively. Therefore, the structure of the fatty acid chain in lipopeptide M1 was considered a linear structure.

With the above data, we performed a detailed NMR analysis to further confirm the lipopeptide structure of the molecule. Inspection of the ^1^H NMR spectra revealed signals corresponding to seven amide groups (-NH-) protons at δH 7.35–8.08 ppm, while the α-amino acid protons of the amino acids at δH 4.47–5.10 ppm (Fig. S5). A doublet at δH 0.83–0.97 ppm indicated a long alkyl chain of the lipopeptide M1 [-(CH_3_)_2_-CH-]. Additional multiples in the up-field region arose from the side-chain protons of the amino acids, and the remaining spectra supported the presence of *β*-hydroxy fatty acid. The ^13^C-NMR spectrum showed strong signals δC at 11.42–17.82, 18.41–58.45, and 170.82-173.89 ppm from methyl, methylene, and carboxyl groups, respectively (Fig. S6), which was consistent with the mass spectrometry results.

**Fig. 5:**
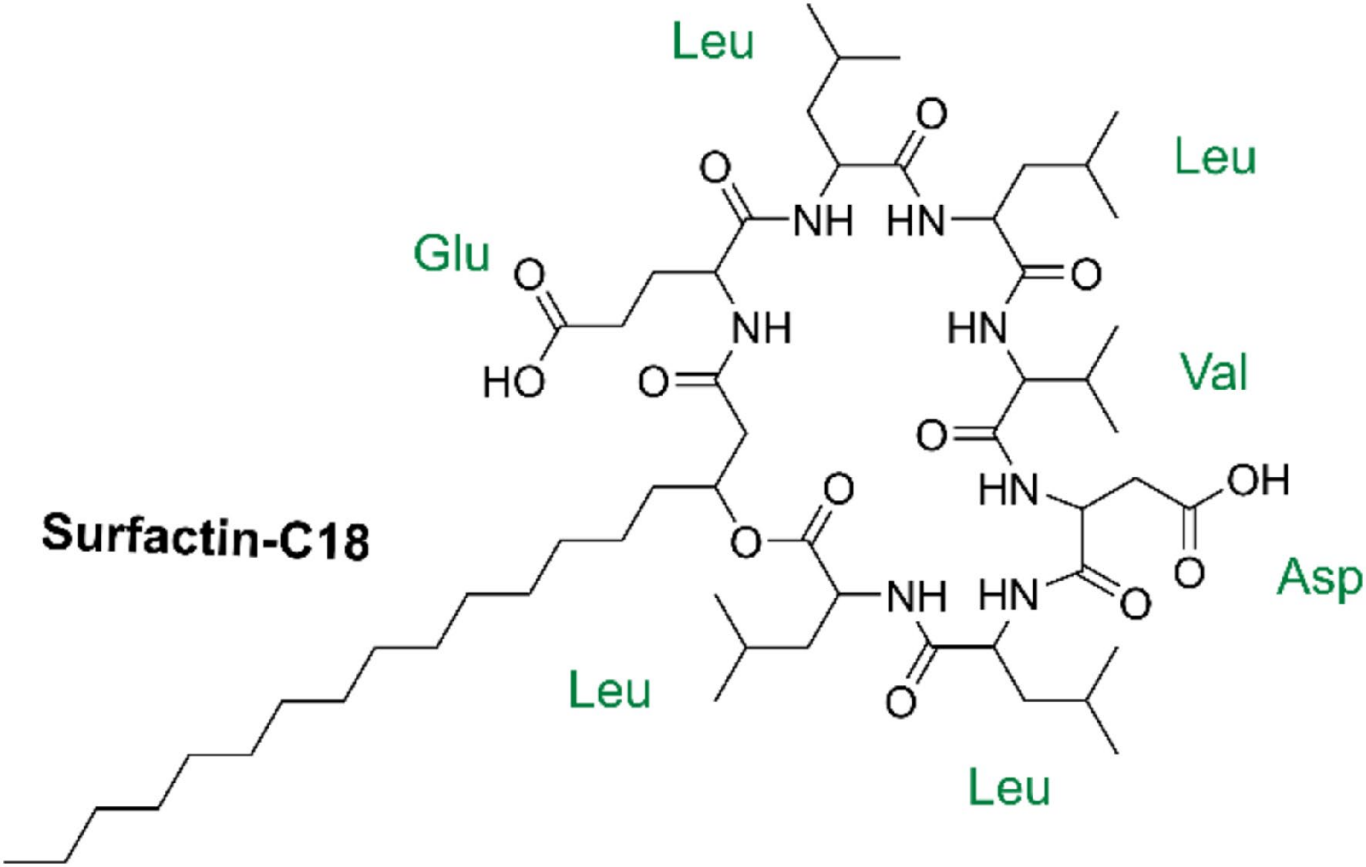
Chemical structure of surfactin-C18 from *B. subtilis* TD7

To sum up, the lipopeptide produced by *B. subtilis* TD7 could be preliminarily concluded as a new surfactin member, the surfactin-C18, which was confirmed by a combination of ESI-MS, LC-MS, GC-MS, ^1^H NMR and ^13^C NMR analyses. The primary structure of the surfactin-C18 incorporates a common peptide loop of seven amino acids (*N*-Glu-Leu-Leu-Val-Asp-Leu-Leu-*C*) containing 18 carbons in the linear fatty acid chain (Fig. 5).

### Surface activity of the surfactin-C18

**Fig. 6:**
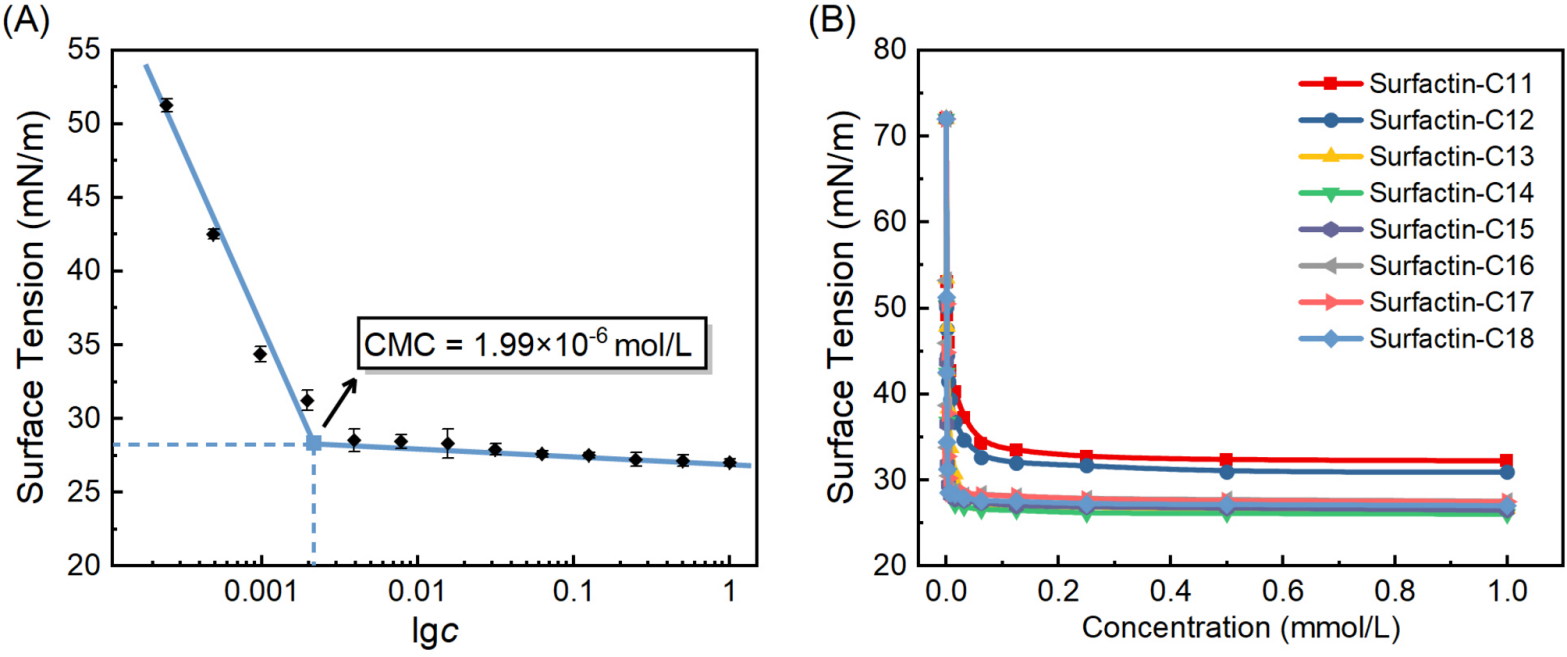
The surface tension isotherm of surfactin-C18 (**A**) and other surfactin homologs (**B**) aqueous solution of various concentrations at 25 ℃

A critical property of biosurfactants is their ability to form micelles and reduce the surface tension or interfacial tension at air/aqueous and oil/aqueous interfaces [40]. As shown in Fig. 6A, with the increasing concentration of surfactin-C18 solution, surface tension at various concentrations can be readily decreased to below 28 mN/m, with the highest tested concentration being 1 mmol/L. According to the breakpoint of the plot of surface tension (*γ*) versus lo*gic*, the critical micelle concentration (CMC) value of surfactin-C18 was 1.99 µmol/L at 25 °C, with a corresponding surface tension at CMC (*γ*_CMC_) of 28.63 mN/m. The adsorption isotherm properties of surfactin-C18 solution were determined using the *γ*-lg*c* fitting curve, including the surface excess concentration (*Γ*), the area occupied by the molecule (A), and the free energy of micellization (Δ*G*) (Table S3). The calculation showed that the maximum surface excess concentration (*Г*_max_) and the corresponding minimum area occupied by one surfactin-C18 molecule (*A*_min_) at the air/liquid interface were estimated to be 1.17 µmol/m^2^ and 141.93 Å^2^, respectively, indicating a compact molecular arrangement of surfactin-C18 at the air/aqueous interface.

**Fig. 7:**
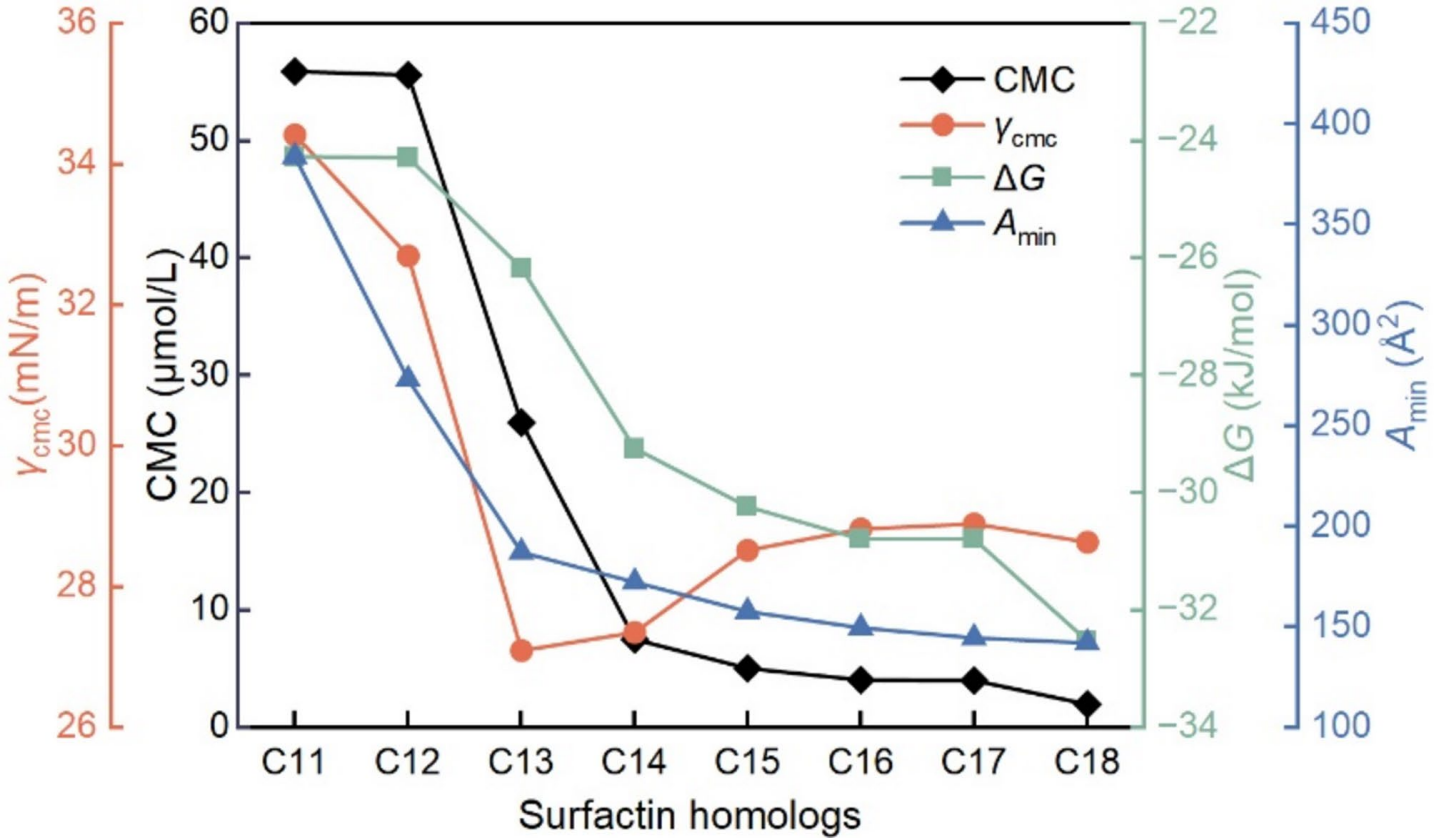
Surface-active and adsorption properties of surfactin-C18 and different fatty acid chains of surfactin homologs

Biosurfactants are distinguished by their amphiphilic structure with a tendency to adsorption at various interfaces, such as air/water and oil/water interfaces[41]. A fundamental parameter for evaluating the performance of biosurfactants is the CMC value, showing the ability to reduce the surface tension at the air/water interface [42]. As biosurfactant concentration increases, surface tension continues to decrease until reaching a concentration termed the CMC value, beyond which the first micelle forms, leading to significant variation in the physicochemical properties of the solution, while above the CMC, the surface tension is extensively independent of concentration [43, 44]. Generally, the biosurfactants with lower CMC values tend to reduce surface tension more effectively at lower concentrations, exhibiting better surface/interface properties in wetting, dispersing, foam-forming, and emulsification [45]. Several methods can be used to determine the CMC value such as surface tension, electrical conductivity and UV-absorption spectroscopy measurements, and in this study, we employed the widely used approach based on the *γ*-lg*c* isotherm [46]. Notably, surfactin-C18 exhibited superior surface activity compared to other surfactin homologs that were previously reported [30]. For comparison, the CMC values of surfactin-C11, C12, C13, C14, C15, C16 and surfactin-C17 are 55.9, 55.6, 26.0, 7.53, 5.04, 4.04 and 4.02 µmol/L, respectively, with corresponding minimum surface tensions of 34.42, 32.70, 27.09, 27.35, 28.52, 28.82 and 28.98 mN/m, respectively (Fig. 7). These values highlight that surfactin-C18 ranks among the most effective surfactins in reducing surface tension. Importantly, when compared to some commercial synthetic surfactants, such as non-ionic surfactant Triton-X 165 (Table S3), surfactin-C18 also exhibited higher efficiency in lowering the surface tension, reinforcing its potential as a high-performance biosurfactant. Additionally, surfactin exhibits the notable ability to maintain low surface tension under various environmental conditions in the pH range from 5 to 13, temperature range from 4 ℃ to 121 ℃ and salinities up to 9%, along with a wide range of bioactive properties, including antimicrobial, antiviral, antibiofilm and hemolysis [47–49]. Consequently, surfactin holds promise as a therapeutic agent, a biosurfactant and a bioremediation agent in potential industrial and therapeutic applications.

The adsorption properties of biosurfactants are mainly related to the chemical structure of the hydrophobic and hydrophilic moiety, including the length of the hydrophobic tail, the type of the peptide moiety, and the overall molecular size [41]. In the case of the structural characteristics, all the above surfactins have the same hydrophilic heads but differ in the length of their hydrophobic tails. Consequently, variations in CMC values can be possibly explained by the difference in the structure and length of the fatty acid chains: the longer hydrophobic chain results in improved surface-active performance. From an isothermal perspective, the biosurfactant concentration in the monolayer at the air/water interface was determined according to the *γ*-lg*c* isotherm and Gibbs adsorption isotherm. As illustrated in Table S3, the adsorption amount of surfactin on the air/aqueous interface increased with the increase in the hydrophobic chain length. The surface area of the monolayer expanded from 0.43 µmol/m^2^ to 1.17 µmol/m^2^, while the free energy decreased from − 24.27 kJ/mol to -32.52 kJ/mol. A possible explanation for this trend is that a longer hydrophobic chain length facilitates interaction at the air/aqueous interface and alters the relative position of the peptide ring, resulting in a reduction of the area occupied by a single molecule. These results clearly indicate that the surface activity of the surfactins enhanced with increasing fatty chain length, which is more conducive to micelle formation. Additionally, the micelle shape was evaluated based on the critical packing parameter (CPP) values, and as expected, the longer hydrophobic chain had an insignificant influence on the micelle structure, which remained spherical micelles (CPP < 1/3). Moreover, previous studies have demonstrated that as the length of the hydrophobic chain in surfactin increased, the diameter of aggregates significantly increased with the formation of larger aggregates [50]. Specifically, as the fatty acid chains of surfactins extend from 12 carbon atoms to 16 carbon atoms, *β*-sheet interaction between the micelles is reinforced, making surfactin more likely to form large aggregates. Despite the promising surface/interface-active properties of surfactin, the scalability of surfactin production remains the major limitation of the industrial application. We previously increased the surfactin yield produced by *B. subtilis TD7* from 0.62 g/L to 2.14 g/L by promoter substitution [51]. In the future, our group will conduct in-depth research on the issue of yield improvement through optimizing the fermentation process, genomic engineering and synthetic biology strategies. Separately, using a microfluidic diffusion platform and molecular dynamics simulation to further clarify the molecular mechanisms at various interfaces. Addressing these challenges would expand the applications of surfactin in bioremediation, microbial enhanced oil recovery, food and pharmaceutical fields.

## Conclusions

In summary, this work highlighted the significant biosynthetic potential of the *Bacillus subtilis* TD7 strain for lipopeptide production through genome analysis and mining. AntiSMASH has been utilized to discover that four of fourteen BGCs are related to lipopeptide biosynthesis, including those for surfactin, fengycin, bacillaene and bacillibactin. In addition, we reported the isolation and structural characterization of a new member of the surfactin family, surfactin-C18, produced by *B. subtilis* TD7. Surfactin-C18 compound was successfully purified using organic extraction followed by preparative HPLC. Its structural features were elucidated using a combination of analytical methods, including ESI-MS, LC-MS, GC-MS and NMR, confirming a heptapeptide ring linked to the longest fatty acid moiety reported to date. Moreover, the surface activity test demonstrated surfactin-C18 exhibited a CMC value of 1.99 µmol/L and a corresponding *γ*_CMC_ of 28.63 mN/m, Compared with other surfactin homologs and commercial surfactants, surfactin-C18 exhibits superior surface-active property. This study reinforces the excellent potential of microorganisms as a valuable resource for novel natural product discovery and emphasizes the importance of continued exploration of *Bacillus* species with a biosynthetic potential. Furthermore, surfactin-C18 presents a promising alternative to traditional synthetic surfactants and other surfactin homologs, with potential applications in microbial enhanced oil recovery, food and cosmetics industry.

## Methods

### Strain and culture conditions

The *Bacillus subtilis* TD7 strain, used in the current study, was obtained from the Daqing oilfield, China [52]. The strain was stored in a 50% (v/v) LB-glycerol solution at -80 ℃ in our laboratory. Initially, a frozen glycerol culture stock was streaked onto a LB agar plate (10 g/L NaCl, 10 g/L peptone, 5 g/L yeast and 2.5 g/L agar powder) for 24 h, and the colony was obtained. Then a single colony was transferred to 100 mL LB liquid medium, and incubated at 37℃ and 180 rpm for 20 h, as the seed culture.

### Fermentation conditions

The 2% (v/v) seed culture was inoculated in 250 mL Erlenmeyer flasks containing 100 mL optimized cultivation medium on rotary shakers at 37 ℃, 200 rpm for 72 h, including 20.000 g/L sucrose, 2.450 g/L KH_2_PO_4_, 3.970 g/L Na_2_HPO_4_, 1.340 g/L NH_4_Cl, 2.125 g/L NaNO_3_, 1.000 g/L yeast, 0.100 g/L MgSO_4_, 0.070 mmol/L CaCl_2_ and 0.100 mmol/L MnCl_2_. For the above medium, pH was adjusted to 7.0-7.5 using 1 mol/L NaOH before autoclaved sterilization. After 72 h cultivation, bacteria were pelleted by centrifugation (Sigma Centrifuge 3K15, Rotor No. 12150) at 8000 rpm for 20 min and the cell-free supernatant was obtained.

### Extraction and purification

Briefly, the lipopeptide extract was obtained by a combination of acid precipitation and ethyl acetate extraction methods. 6 mol/L HCl was titrated to adjust the pH value of the supernatant to pH < 2.0, and the suspension was left at 4 ℃ overnight. The suspension was centrifuged at 8000 rpm for 10 min to get the acid precipitation, by washing three times with deionized water. Subsequently, the precipitation was lyophilized until dry and then extracted with ethyl acetate three times to obtain lipopeptide extracts. The crude extracts were suspended in 100% methanol and injected into a reverse-phase high-performance liquid chromatography (RP-HPLC) (JASCO, Japan) after filtering through a 0.22 μm filter membrane. Mobile phase A was 100% methanol and mobile phase B was ultra-pure water with 0.05% (v/v) TFA. The proportion of the mobile phases was controlled via the following gradient program: 0–90 min, 90% A and 90–100 min, 100% A. The elution fraction of peaks was collected manually and freeze-dried to perform structural analysis.

### Genome sequencing and assembly

Genomic DNA of *Bacillus subtilis* TD7 from a pure culture using the PowerSoil DNA Isolation Kit (MO BIO, USA) and the genome sequencing was performed using Illumina Hiseq 2000 sequencer according to the standard protocol, including DNA extraction, DNA sample quality testing, library construction, library purification, library detection, library quantification, generation of sequencing clusters, and up-sequencing. After obtaining the whole genome sequence, the bioinformatic work was mainly performed based on three databases: COG (Clusters of Orthologous Groups, http://www.ncbi.nlm.nih.gov/COG/), GO (Gene Ontology, http://www.geneontology.org) and KEGG (Kyoto Encyclopedia of Genes and Genomes, http://www.genome.jp/kegg/). BGCs responsible for the biosynthesis of secondary metabolites were identified using the antiSMASH webserver [53].

### Structural elucidation

Generally, the eluted fraction was obtained by RP-HPLC, and a detailed structural characterization was performed by a diverse range of analytical methods as previously described [17]. The molecular mass of the lipopeptide extract was determined by high-resolution electrospray ionization mass spectrometry (ESI-MS) (Water, America) at the Analysis and Test Centre, East China University of Science and Technology. The *m/z* values were measured from 100 to 2000. Additionally, the lipopeptide extract was hydrolyzed with 1 mL 6 mol/L HCl at 90 ℃ for 20 h in a stoppered test tube, followed by the removal of excess solvent with air blow at 60 ℃. The fatty acid residue was labeled with 0.5 mL of acetonitrile-BSTFA (*N*, *O*-bis (trimethylsilyl)-trifluoroacetamide, 3:2, v/v), and heated at 60 ℃ for 20 min. The labeled sample was injected into GC-MS prior to adding 1 mL acetonitrile to dissolve. Moreover, the analysis of the amino acids was also carried out by GC-MS after acid hydrolysis and trimethylsilylation. The GC-MS analysis was performed on a 6890 GC system (Agilent, USA) coupled with a 5975 MSD (Agilent, USA) and equipped with an HP-5MS capillary column (30 m × 0.25 mm × 0.25 μm). The sample after silylation was injected and analyzed with EI operating at 70 eV and used a source temperature of 230 °C. The column oven temperature was held initially at 60 °C for 3 min, then increased to 250 °C at a rate of 10 °C/min, and held at 250 °C for 5 min. Other operating conditions were as follows: helium carrier gas, 99.999%; flow rate, 1.0 mL/min; injector temperature, 250 °C; injector volume, 2.0 µL; and split ratio, 20:1. All NMR spectra were recorded at 298 K on a Bruker Avance 600 MHz with 1.0 mL CDCl_3_ as a solvent.

### Surface property assay

Different amounts of lipopeptide were completely dissolved in water to form a series of solutions with concentrations ranging from 0.01 to 1% (w/v). The surface tension of these solutions was measured at 25 °C using a DCAT 21 tensiometer (Dataphysics, Germany). The critical micelle concentration (CMC) value and the surface tension at CMC (*γ*_CMC_) were calculated according to the plot of surface tension versus lipopeptide concentration [54]. The derivative of the equilibrium surface tension curve (d*γ*/dlg*c*) at the CMC can be used to estimate surface properties, including the maximum surface excess concentration (*Г*_max_) and the minimum area occupied by a surfactin molecule (*A*_min_) at the air/liquid interface [55]. The calculation of the above parameters was carried out using the following equations:

$${\it{\Gamma} _{{\rm{max}}}}{\rm{ = }}\, - {{\rm{1}} \over {{\rm{2}}{\rm{.303R}}T}}\left( {{{{\rm{d}}\gamma } \over {{\rm{dlgc}}}}} \right)$$

$$\,{{\rm{A}}_{{\rm{min}}}}{\rm{ = }}\,{{\rm{1}} \over {{\rm{N}}{\it{\Gamma} _{{\rm{max}}}}}}$$

$$\,\Delta G = {\rm{R}}T{\rm{lnc}}$$

Where *γ* is the surface tension, R is the universal gas constant (8.314 J mol^− 1^ K^− 1^), *T* is the absolute temperature, and *c* is the surfactant concentration, and N is the Avogadro number (6.022 × 10^23^).

### Statistical analysis

All experiments were conducted in at least three independent biological replicates, with each sample measured in triplicate for each assay. Data are presented as mean ± standard deviation (SD) using OriginPro 2023. The presented images are representative of multiple independent experiments that yielded consistent results.

## Electronic supplementary material

Below is the link to the electronic supplementary material.

Supplementary Material 1

## Data Availability

No datasets were generated or analysed during the current study.

## Abbreviations

BGC: Biosynthetic gene clusters
CDS: Protein-coding genes
NRPSs: Nonribosomal peptide synthetases
COG: Clusters of Orthologous Groups
GO: Gene Ontology
KEGG: Kyoto Encyclopedia of Genes and Genomes
ESI-MS: Electrospray ionization mass spectrometry
RP-HPLC: Reverse-phase high-performance liquid chromatography
LC-MS: Liquid chromatography-mass spectrometry
DHT: The double hydrogen transfer
TLC: Thin-layer chromatography
GC-MS: Gas chromatography-mass spectrometer
Leu: Leucine
Asp: Aspartic acid
Glu: Glutamic acid
Val: Valine
NMR: Nuclear magnetic resonance
CMC: Critical micelle concentration
CPP: Critical packing parameter
MEOR: Microbial enhanced oil recovery
