# Supplementary material for: Genome mining, structural elucidation and surface-active property of a new lipopeptide from Bacillus subtilis
Source: Microb Cell Fact. 2025 May 14;24:106. doi: 10.1186/s12934-025-02723-y (PMC12076922; doi:10.1186/s12934-025-02723-y)
Supplement: Supplementary file 1 — Supplementary Material 1 [file 12934_2025_2723_MOESM1_ESM.docx]

***Additional file for***

**Genome mining, structural elucidation and surface-active property of a new lipopeptide from *Bacillus subtilis***

Wan-Qi Qin^1^, Yi-Fan Liu ^1,2^, Lei Zhou^1^, Jin-Feng Liu^3^, Dan Fei^4^, Ke-Heng Xiang^1^, Shi-Zhong Yang^1,2^, Ji-Dong Gu^5^, Bo-Zhong Mu^1,2,*^

^1^ State Key Laboratory of Bioreactor Engineering and School of Chemistry and Molecular Engineering, East China University of Science and Technology, Shanghai 200237, P.R. China.

^2^ Shanghai Collaborative Innovation Center for Biomanufacturing Technology, Shanghai 200237, P.R. China

^3^ Daqing Huali Biotechnology Co., Ltd, Daqing, Heilongjiang, 163511, P. R. China.

^4^ Institute of Quality Safety and Standards of Agricultural Products, Jiangxi Academy of Agricultural Sciences, Nanchang, Jiangxi, 330200, P. R. China.

^5^ Environmental Science and Engineering Group, Guangdong Technion Israel Institute of Technology, 241 Daxue Road, Shantou, Guangdong 515063, P.R. China

*Email: bzmu@ecust.edu.cn

**FIGURES**


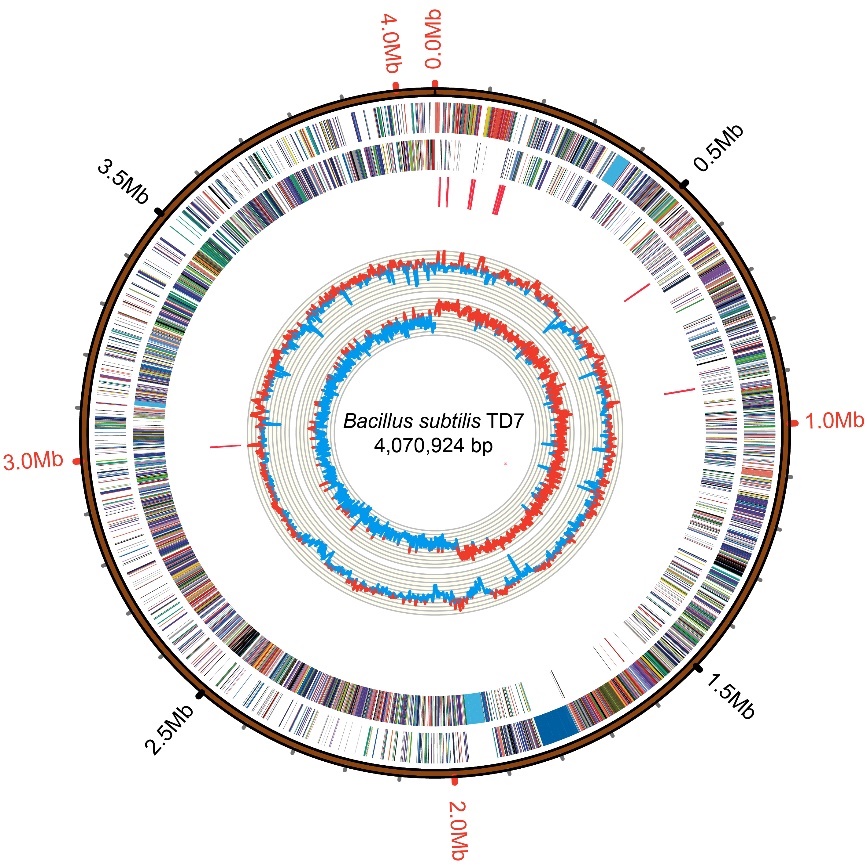


**Figure S1.** The circular genome map *B. subtilis* TD7 with different features. (A) The circular view of the genome. From the outermost to the centre, ring 1 for scale marks of the genome, ring 2 for protein-coding genes on the forward strand, ring 3 for protein-coding genes on the reverse strand, ring 4 for tRNA (black) and rRNA (red) genes on the forward strand, ring 5 for tRNA (black) and rRNA (red) genes on the reverse strand, ring 6 for GC content, ring 7 for GC skew. Protein-coding genes are colour-coded according to their COG categories.


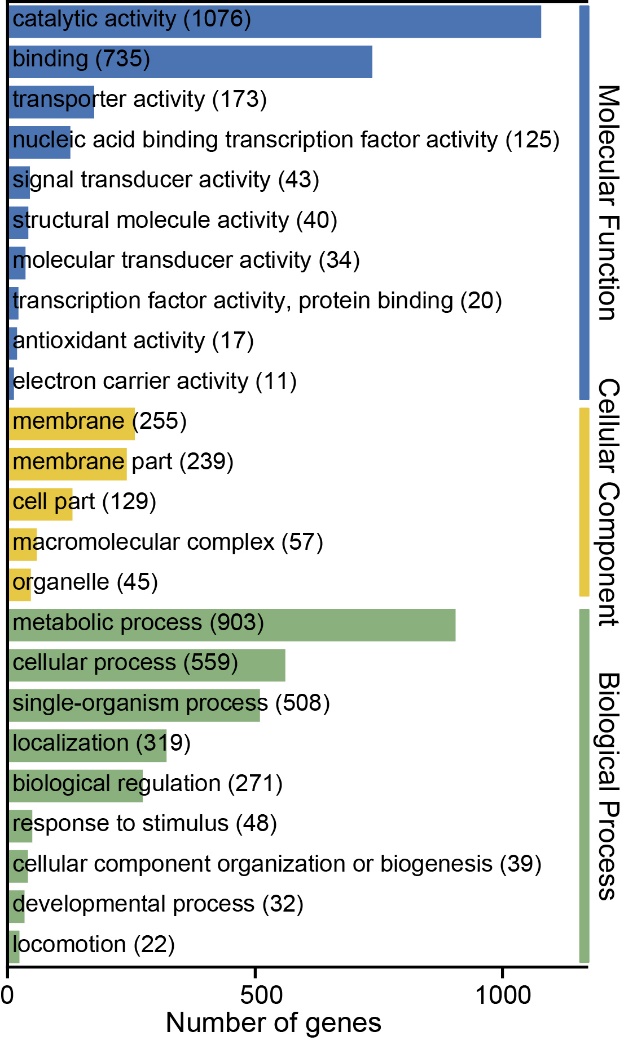


**Figure S2**. GO annotation classification of *B. subtilis* TD7 genome.


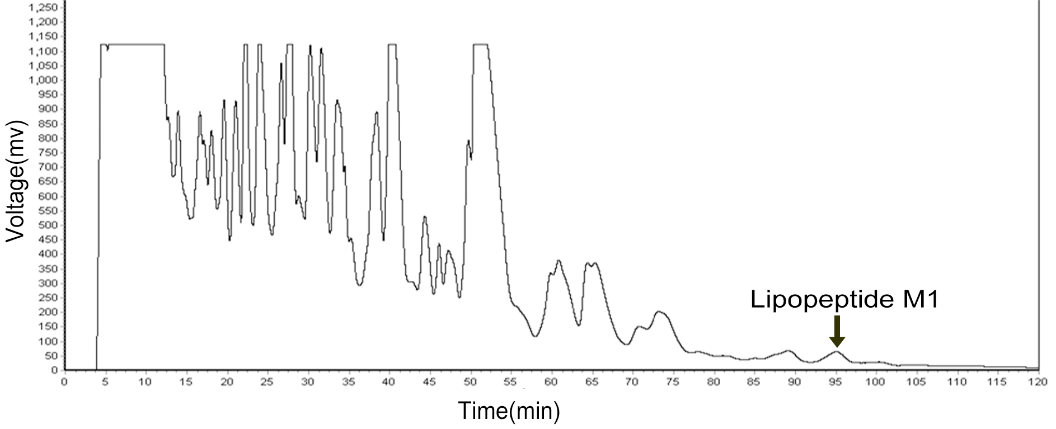
**Figure S3.** Isolation and purification of lipopeptide extract by RP-HPLC.


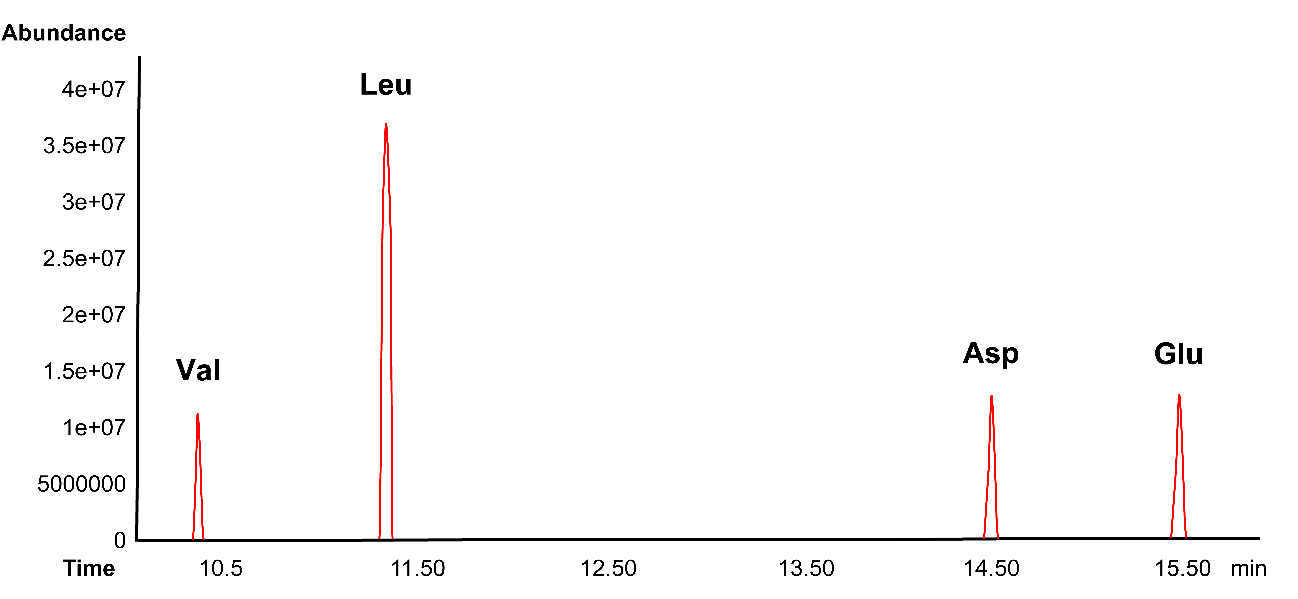
**Figure S4.** The total ion and mass chromatograms of the derivatives of amino acids in lipopeptide M1 after trimethylsilylation.


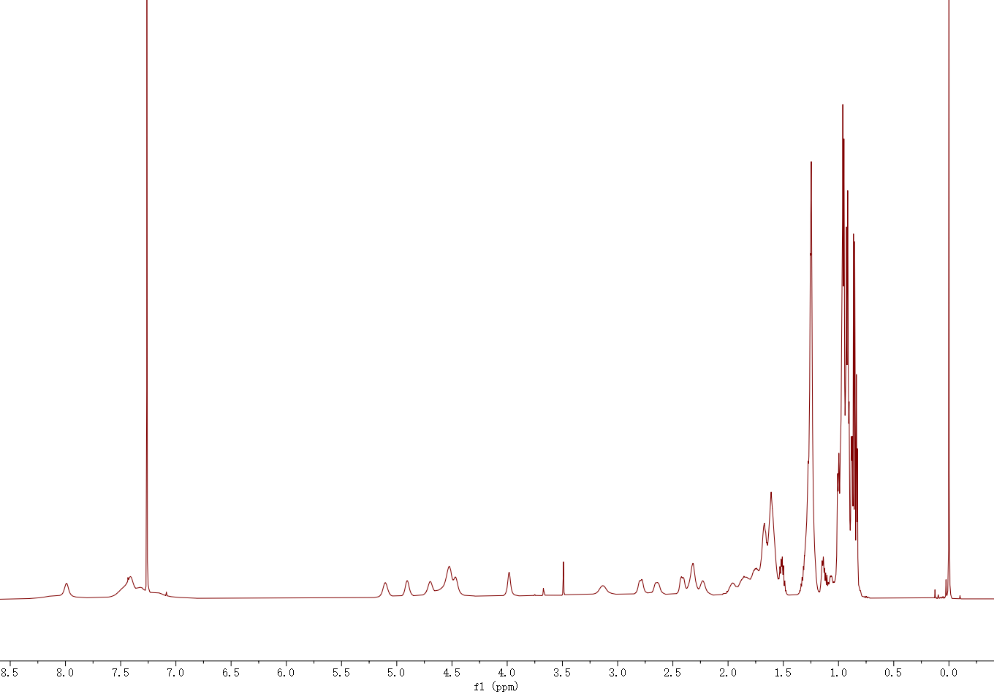


**Figure S5.** ^1^H NMR spectrum (CDCl_3_) of surfactin-C18.


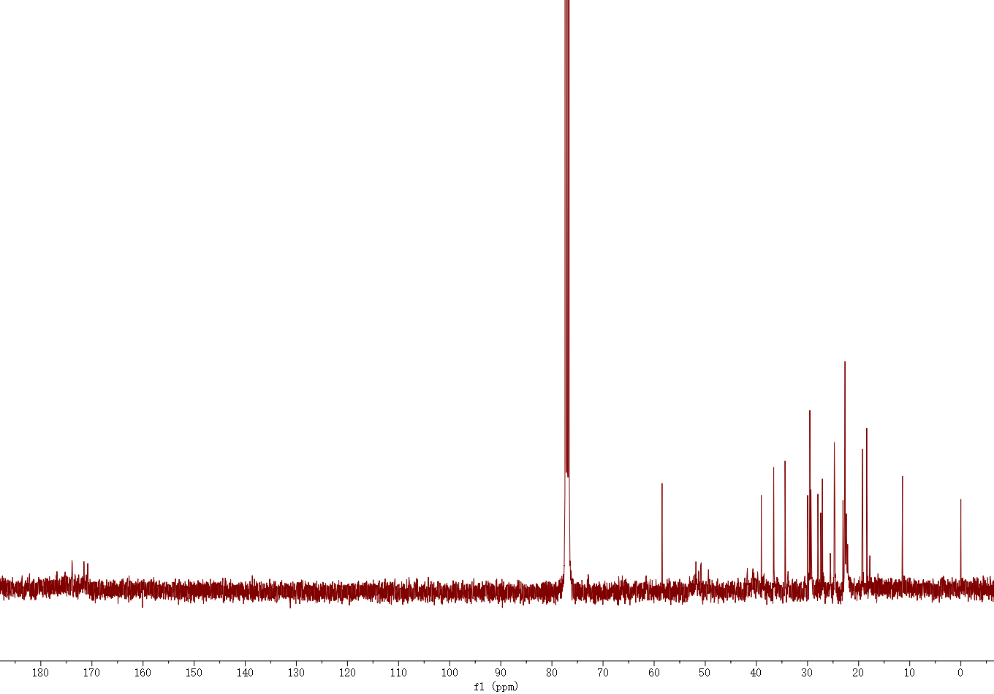


**Figure S6.** ^13^C NMR spectrum (CDCl_3_) of surfactin-C18.

**TABLES**

**Table S1**. Genome statistics of *B. subtilis* TD7.

| Genomic Feature | Value |
| --- | --- |
| Genome size (bp) | 4070924 |
| GC content (%) | 43.81% |
| Protein-coding genes | 4236 |
| Protein-coding regions (bp) | 3613914 |
| rRNA genes | 30 |
| sRNA genes | 11 |

**Table S2**. Detailed secondary metabolite clusters present in genome of *Bacillus subtilis* TD7.

| Region | Type | Compound |  | Similarity |
| --- | --- | --- | --- | --- |
| 1 | NRPS | Surfactin | C52H91N7O13 | 82% |
| 2 | terpene |  |  | / |
| 3 | lanthipeptide-class-i |  |  | / |
| 4 | NRPS, PKS-like, T3PKS, transAT-PKS | Bacillaene | C34H50N2O6 | 100% |
| 5 | NRPS, betalactone | Fengycin | C72H110N12O20 | 100% |
| 6 | terpene | 1-carbapen-2-em-3-carboxylic acid | / | / |
| 7 | T3PKS |  |  | 16% |
| 8 | NRP-metallophore, NRPS | Bacillibactin | C39H42N6O18 | 100% |
| 9 | lanthipeptide-class-i | Subtilin | C12H20N2O4 | 92% |
| 10 | CDPS | Pulcherriminic acid | C12H20N2O4 | 100% |
| 11 | sactipeptide | Subtilosin A | C129H208N36O41S3 | 100% |
| 12 | other | bacilysin | C12H18N2O5 | 100% |
| 13 | RiPP-like |  |  | / |
| 14 | epipeptide | thailanstatin A | C28H41NO9 | 10% |

**TABLE S3**. Adsorption parameters of surfactin-C18 and other surfactin homologs at the air-aqueous solution interface.

| Compound^*^ | CMC  (µmol/L) | *γ_cmc_*  (mN/m) | Δ*G*  (kJ/mol) | *Γ_max_*  (µmol/m^2^) | *A_min_^a^* (Å^2^) | *V_c_^b^*  (nm^3^) | *I_c_^d^*  (nm) | *CPP* | Shape |
| --- | --- | --- | --- | --- | --- | --- | --- | --- | --- |
| SF-C11 | 55.9 | 34.42 | -24.27 | 0.43 | 383.77 | 242.6 | 11.62 | 0.05 | Sphere |
| SF-C12 | 55.6 | 32.70 | -24.29 | 0.61 | 273.01 | 269.5 | 12.89 | 0.08 |  |
| SF-C13 | 26.0 | 27.09 | -26.17 | 0.89 | 187.16 | 296.4 | 14.15 | 0.11 |  |
| SF-C14 | 7.53 | 27.35 | -29.24 | 0.96 | 172.24 | 323.3 | 15.42 | 0.12 |  |
| SF-C15 | 5.04 | 28.52 | -30.24 | 1.06 | 157.54 | 350.2 | 16.68 | 0.13 |  |
| SF-C16 | 4.04 | 28.82 | -30.79 | 1.12 | 149.67 | 377.1 | 17.95 | 0.14 |  |
| SF-C17 | 4.02 | 28.89 | -30.79 | 1.15 | 144.58 | 404.0 | 19.21 | 0.15 |  |
| SF-C18 | 1.99 | 28.63 | -32.52 | 1.17 | 141.93 | 430.9 | 20.48 | 0.15 |  |
| Triton X-165 | 541 | 39.50 | -28.10 | 2.12 | 78.32 | / | / | / | / |

^*^ SF refers to surfactin. Triton X-165 is a non-ionic surfactant and the related parameters were taken from the literature.^29^

^a^ Head group area was calculated from the *γ*-lg*c* slope

^b^ Hydrophobic chain volume was calculated using $\text{V}_{\text{c}}\text{ = 27.4 + 26.9}\text{n}_{\text{c}}$

^c^ Hydrophobic chain length was calculated using $\text{I}_{\text{c}}\text{ = 1.5 + 1.265}\text{n}_{\text{c}}$

^d^ Critical packing parameter (CPP) was calculated using $\text{CPP}\text{ =}\text{ }{\text{V}_{\text{c}}}/{\text{(}\text{I}_{\text{c}}\text{A}\text{)}}$
